# Immunochemical analysis of cathepsin B in lung tumours: an independent prognostic factor for squamous cell carcinoma patients

**DOI:** 10.1038/sj.bjc.6690723

**Published:** 1999-10

**Authors:** B Werle, H Lötterle, U Schanzenbächer, T T Lah, E Kalman, K Kayser, H Bülzebruck, J Schirren, M Krasovec, J Kos, E Spiess

**Affiliations:** Thoraxklinik Heidelberg-Rohrbach, Abteilung für Klinische Chemie und Bakteriologie, Abteilung für Pathologie, Statistische Dokumentation und, Chirurgie, Heidelberg, D-69126, Germany; National Institute of Biology, Ljubljana, SLO-61111, Slovenia; Department of Pathology, University Medical School of Pecs, Pecs, H-7643, Hungary; Department of Biochemistry and Molecular Biology, Jozef-Stefan-Institute, Ljubljana, SLO-61111, Slovenia; KRKA d.d. R&D Division, Department of Biochemical Research and Drug Design, Novo, Mesto, SLO-8000, Slovenia; Biomedizinische Strukturforschung, Deutsches Krebsforschungszentrum, Heidelberg, D-69120, Germany

**Keywords:** cathepsin B, prognosis, non-small cell lung cancer, squamous cell carcinoma

## Abstract

In order to evaluate the possible role of the proteolytic enzyme cathepsin B (cath B) in human non-small cell lung cancer (NSCLC) we examined cath B concentrations (cath B_c_) and activities (cath B_A_) in homogenates of 127 pairs of lung tumour tissues and corresponding non-tumourous lung parenchyma. Total cath B activity (cath B_AT_) and enzymatic activity of the fraction of cath B, which is stable and active at pH 7.5 (cath B_A7.5_) were determined by a fluorogenic assay using synthetic substrate Z-Arg-Arg-AMC. The immunostaining pattern of cath B was determined in 239 lung tumour tissue sections, showing the presence of the enzyme in tumour cells (cath B_T-I_) and in tumour-associated histiocytes (cath B_H-I_). The median levels of cath B_AT_, cath B_A7.5_ and cath B_C_ were 5.6-, 3.2- and 9.1-fold higher (*P* < 0.001), respectively, in tumour tissue than in non-tumourous lung parenchyma. Out of 131 tissue sections from patients with squamous cell carcinoma (SCC), 59.5% immunostained positively for cath B, while among the 108 adenocarcinoma (AC) patients 48.2% of tumours showed a positive reaction. There was a strong relationship between the levels of cath B_AT_, cath B_A7.5_, cath B_C_ and cath B_T-I_ in the primary tumours and the presence of lymph node metastases. Significant correlation with overall survival was observed for cath B_T-I_ and cath B_A7.5_ (*P* < 0.01 and *P* < 0.05, respectively) in patients suffering from SCC. In these patients positive cath B in tumour cells (cath B_T-I_) and negative cath B in histiocytes (cath B_H-I_) indicated significantly shorter survival rate compared with patients with negative cath B_T-I_ and positive cath B_H-I_ (*P* < 0.0001). In contrast, in AC patients, both, positive cath B_T-I_ and positive cath B_H-I_, indicated poor survival probability (*P* < 0.014). From these results we conclude that the proteolytic enzyme cath B is an independent prognostic factor for overall survival of patients suffering from SCC of the lung. © 1999 Cancer Research Campaign


*British Journal of Cancer* (1999) **81**(3), 510–519
© 1999 Cancer Research Campaign
Article no. bjoc.1999.0723

### Immunochemical analysis of cathepsin B in lung tumours: an independent prognostic factor for squamous cell carcinoma patients

**B Werle[1], H Lötterle[1], U Schanzenbächer[1], TT Lah[5], E Kalman[6], K Kayser[2], H Bülzebruck[3], J Schirren[4], M Krasovec[7], J Kos[7,8] and E Spiess[9]**

[1]Thoraxklinik Heidelberg-Rohrbach, Abteilung für Klinische Chemie und Bakteriologie, [2]Abteilung für Pathologie, [3]Statistische Dokumentation und [4]Chirurgie, D-69126 Heidelberg, Germany; [5]National Institute of Biology, SLO-61111 Ljubljana, Slovenia; [6]Department of Pathology, University Medical School of Pecs, H-7643 Pecs, Hungary; [7]Department of Biochemistry and Molecular Biology, Jozef-Stefan-Institute, SLO-61111 Ljubljana, Slovenia; [8]KRKA d.d., R&D Division, Department of Biochemical Research and Drug Design, SLO-8000 Novo Mesto, Slovenia; [9]Biomedizinische Strukturforschung, Deutsches Krebsforschungszentrum, D-69120, Heidelberg, Germany

**Summary** In order to evaluate the possible role of the proteolytic enzyme cathepsin B (cath B) in human non-small cell lung cancer (NSCLC) we examined cath B concentrations (cath $B_c$) and activities (cath $B_A$) in homogenates of 127 pairs of lung tumour tissues and corresponding non-tumourous lung parenchyma. Total cath B activity (cath $B_{AT}$) and enzymatic activity of the fraction of cath B, which is stable and active at pH 7.5 (cath $B_{A7.5}$) were determined by a fluorogenic assay using synthetic substrate Z-Arg-Arg-AMC. The immunostaining pattern of cath B was determined in 239 lung tumour tissue sections, showing the presence of the enzyme in tumour cells (cath $B_{T-I}$) and in tumour-associated histiocytes (cath $B_{H-I}$). The median levels of cath $B_{AT}$, cath $B_{A7.5}$ and cath $B_C$ were 5.6-, 3.2- and 9.1-fold higher ($P < 0.001$), respectively, in tumour tissue than in non-tumourous lung parenchyma. Out of 131 tissue sections from patients with squamous cell carcinoma (SCC), 59.5% immunostained positively for cath B, while among the 108 adenocarcinoma (AC) patients 48.2% of tumours showed a positive reaction. There was a strong relationship between the levels of cath $B_{AT}$, cath $B_{A7.5}$, cath $B_C$ and cath $B_{T-I}$ in the primary tumours and the presence of lymph node metastases. Significant correlation with overall survival was observed for cath $B_{T-I}$ and cath $B_{A7.5}$ ($P < 0.01$ and $P < 0.05$, respectively) in patients suffering from SCC. In these patients positive cath B in tumour cells (cath $B_{T-I}$) and negative cath B in histiocytes (cath $B_{H-I}$) indicated significantly shorter survival rate compared with patients with negative cath $B_{T-I}$ and positive cath $B_{H-I}$ ($P < 0.0001$). In contrast, in AC patients, both, positive cath $B_{T-I}$ and positive cath $B_{H-I}$, indicated poor survival probability ($P < 0.014$). From these results we conclude that the proteolytic enzyme cath B is an independent prognostic factor for overall survival of patients suffering from SCC of the lung. © 1999 Cancer Research Campaign

**Keywords:** cathepsin B; prognosis; non-small cell lung cancer; squamous cell carcinoma

In recent years several studies have aimed at revealing the pathobiochemical functions of cathepsin B (cath B) in tumours and have led to the hypothesis that the enzyme is relevant for the progression of tumours, i.e. metastasis (reviewed by Berquin and Sloane, 1995; Elliot and Sloane, 1996; Lah and Kos, 1998; Yan et al, 1998). There are a number of evidences, supporting this hypothesis: the levels of cath B in tumours were elevated above the levels in adjacent normal tissue as demonstrated for example in lung carcinoma (Trefz et al, 1989; Krepela et al, 1990; Sukoh et al, 1994*a*, 1994*b*; Ebert et al, 1994; Ledakis et al, 1996; Werle et al, 1997*a*, 1997*b*), breast carcinoma (Lah et al, 1992, 1995*a*), colon carcinoma (Shuja et al, 1991), head and neck carcinoma (Budihna et al, 1996), oesophageal carcinoma (Hughes et al, 1998) and glioma (Rempel et al, 1994; Mikkelsen et al, 1995). Elevated levels of the enzyme were also found in sera of tumour patients (Leto et al, 1997; Kos et al, 1997, 1998). Cath B was found concentrated at the invasive edges of colon carcinoma (Emmert-Buck et al, 1994). In vitro, tumour cells were found to be able to target cath B to the plasma membrane of tumour cells and to focal adhesion points relevant for motility to a greater extent than in normal cells (Rozhin et al, 1987; Sloane et al, 1987, 1994; Erdel et al, 1990; Strohmaier et al, 1997). Increased secretion of the inactive proform or the active enzyme (Werle et al, 1994, 1996) was sensitive to external stimuli, i.e. changes in pH or signal mediator 12(S)-HETE (Rozhin et al, 1994; Honn et al, 1994; Ulbricht et al, 1996). The balance between the enzyme and its physiological inhibitors seems to be disturbed in tumours (Knoch et al, 1994; reviewed by Calkins and Sloane, 1995; Lah et al, 1997) and finally, in vitro concerted inhibition of cath B and collagenases led to the most effective reduction of extracellular matrix degradation (Stonelake et al, 1997).

The problem in defining a role of cath B in tumour progression arises from the fact that the enzyme is coded by a housekeeping gene, present in all, tumour and normal cells. Moreover, cath B was also found in tumour surrounding stromal cells and particularly in activated monocytes, which produce increased levels of cath B (Lah et al, 1995*b*). The interplay between tumour and tumour surrounding cells, including autocrine and paracrine mechanisms, resulting in an overproduction of cath B by different cell types, is still not well understood (Opdenakker et al, 1992). However, the appearance of the enzyme in tumours, if associated with malignancy and metastasis may be useful in prediction of

*Received 28 October 1998*
*Revised*
*Accepted 4 March 1999*

*Correspondence to:* B Werle survival probability of patients with lung cancer after surgical treatment and thereby improving the effectiveness of post-operative therapy.

Higashiyama et al (1991, 1993) demonstrated increased expression of cath B in lung adenocarcinomas. Sukoh et al (1994*a*, 1994*b*) performed retrospective immunohistochemical studies, which showed that patients with tumours, strongly positive for cath B, had a significantly shorter overall survival compared with patients, which were negative for cath B. The authors recommended cath B as an independent prognostic marker in non-small-cell lung cancer (NSCLC), similar as concluded also by Inoue et al (1994). We have also demonstrated a correlation between high native cath B activity at pH 6.0 (cath $B_{AT}$) and shorter survival probability of lung cancer patients (Ebert et al, 1994). Recently, even more significant correlation with shorter survival was found for high activity of cath B-fraction, which was stable and active at pH 7.5 (cath $B_{A7.5}$) (Werle et al, 1997*b*). However, these studies were performed on relatively small population of patients and by a unifactorial methodological approach.

Here, we present an extended study on cath B activity and protein concentration in comparison with immunohistochemical (IHC) analysis of lung tumour sections of the same tumours. IHC analysis would have an advantage over immunochemical and activity analysis of tumour homogenates, due to the simplicity and less tumour tissue needed and would also allow for the discrimination between tumour cell and histiocytes associated cath B. Taken all results together, we demonstrated that IHC analysis of cath B offers a simple and independent prognostic factor for overall survival of patients with squamous cell carcinoma (SCC) of the lung.

### PATIENTS, MATERIALS AND METHODS

### Patients

We investigated two groups of patients for the levels of cath B. In the first group, comprising randomly selected 127 patients (15–81 years of age, mean 58.9 years, 104:23 male:female) we determined enzymatic activities and protein concentration, using an enzyme-linked immunosorbent assay (ELISA). Immunohistochemical (IHC) analysis was performed in 92 out of these 127 patients and in an additional 147 patients (38–87 years of age, mean 61.0 years, 114:33 male:female), comprising therefore a total of 239 samples for IHC analysis. Cell typing of lung cancer was based on the

**Table 1** Cathepsin B in lung tumour homogenates

| | | Cath $B_{AT}$ (μEU mg$^{-1}$ protein$^{-1}$) | | | | Cath $B_{A7.5}$ (μEU mg$^{-1}$ protein$^{-1}$) | | | | Cath $B_C$ (ng mg$^{-1}$ protein$^{-1}$) | | |
|---|---|---|---|---|---|---|---|---|---|---|---|---|
| | *n* | Tumour median (5%, 95%) | Normal median (5%, 95%) | Tu/Lu median | *n* | Tumour median (5%, 95%) | Normal median (5%, 95%) | Tu/Lu median | *n* | Tumour median (5%, 95%) | Normal median (5%, 95%) | Tu/Lu median |
| Primary tumours (total) vs normal lung tissue (total) | 127 | 1237[a] (286, 2597) | 223 (51, 1081) | 5.55 | 124 | 394[a] (33, 592) | 125 (35, 592) | 3.15 | 127 | 1215[a] (357, 2692) | 133 (40, 367) | 9.14 |
| – Squamous cell carcinoma (SCC) | 68 | 1168[a] (234, 2724) | 235 (47, 1058) | 4.97 | 65 | 330[a] (17, 1856) | 125 (29, 577) | 2.64 | 68 | 1213[a] (479, 2702) | 121 (29, 312) | 10.00 |
| – Adenocarcinoma (AC) | 59 | 1282[a] (439, 2585) | 200 (57, 1142) | 6.41 | 59 | 397[a] (124, 1772) | 205 (37, 636) | 1.94 | 59 | 1217[a] (270, 2682) | 135 (56, 376) | 9.00 |
| pT1 | 20 | 1184 (248, 4772) | | | 20 | 308 (14, 1856) | | | 20 | 1233 (300, 2424) | | |
| pT2 | 75 | 1278 (321, 3100) | | | 75 | 414 (55, 1721) | | | 75 | 1210 (383, 2702) | | |
| pT3 | 20 | 1168 (219, 2547) | | | 20 | 396 (9, 1893) | | | 20 | 1379 (644, 2283) | | |
| pT4 | 12 | 1094 (560, 2388) | | | 12 | 323 (39, 1624) | | | 12 | 1049 (593, 3585) | | |
| pN0 | 43 | 1598 (321, 3807) | | | 43 | 292 (30, 1749) | | | 43 | 1147 (298, 2283) | | |
| pN1 | 43 | 1124 (218, 2149) | | | 43 | 414 (17, 1624) | | | 43 | 1345 (574, 3125) | | |
| pN2 | 27 | 970 (514, 2597) | | | 27 | 443 (117, 1893) | | | 27 | 1054 (529, 2682) | | |
| pN3 | 14 | 1819 (353, 2508) | | | 14 | 760 (190, 1772) | | | 14 | 1764 (864, 3382) | | |
| pTNM I | 35 | 1598 (321, 6820) | | | 35 | 301 (30, 1856) | | | 35 | 1147 (270, 2615) | | |
| pTNM II | 24 | 1224 (218, 2149) | | | 24 | 428 (17, 2248) | | | 24 | 1388 (616, 2702) | | |
| pTNM IIIa | 36 | 1008 (271/2597) | | | 36 | 352 (27/1893) | | | 36 | 1222 (556/2283) | | |
| pTNM IIIb | 19 | 1145 (353, 2388) | | | 19 | 733 (39, 1772) | | | 19 | 2048 (593, 3585) | | |
| pTNM IV | 13 | 1423 (898/2573) | | | 13 | 408 (59/1238) | | | 13 | 1177 (707/1636) | | |

[a]Cathepsin B values were significantly increased in tumour tissue (Tu) compared to its corresponding lung parenchyma (Lu), $P < 0.001$.

© 1999 Cancer Research Campaign 

predominant cell type and was done according to the WHO protocol (World Health Organization, 1981). The tumour disease stage (pTNM) was classified according to the international staging system (Hermanek and Sobin, 1987). All patients included in this study were subjected to surgery, also 51 patients with pTNM IIIb and those 21 patients with pTNM IV, who undergo curative surgery due to incorrect preoperative clinical staging (cTNM) or due to individual favourable conditions. None of these patients were exposed to radiation therapy and received chemotherapy prior to surgery. After curative surgery patients in low stages were kept under surveillance, while patients in high stages additionally received adjuvant or palliative chemotherapy and/or radiation therapy (Manegold and Drings, 1998; Schraube et al, 1998).

### Cathepsin B activity (cath $B_{AT}$, cath $B_{A7.5}$) assays

Both assays were performed exactly as described in detail earlier (Werle et al, 1997*a*, 1997*b*). Total cath $B_{AT}$ activity was measured with the substrate Z-Arg-Arg-AMC at pH 6.0. To receive the cath B fraction, which is stable and active at pH 7.5 (cath $B_{A7.5}$), tissue homogenates were preincubated for 60 min at pH 7.5 and residual cath B activity was determined at the same pH. Specificity of the cath B fraction, which is stable and active at pH 7.5 (cath $B_{A7.5}$) was verified by using the synthetic inhibitor CA-074.

### Cathepsin B ELISA

Human cath B protein (cath $B_C$) in lung tissue homogenates was analysed using an ELISA (KRKA d.d., Novo mesto, Slovenia). The components were purified and characterized and the test was optimized as described (Kos et al, 1995, 1997). The antibodies used for cath B ELISA recognize precursors, the mature form of the enzyme as well as enzyme-inhibitor complexes (Kos et al, 1995). A microplate reader (SLT Rainbow, Austria) was used to measure absorbance. Cath B determined in ng $ml^{-1}$ was standardized in ng $mg^{-1}$ total protein of the tissue homogenates.

### Determination of protein concentration

Protein concentration was determined according to Bradford (1976). Bovine serum albumin was used as a standard.

### Immunohistochemistry

Sections (3–5 μm) from formalin-fixed, paraffin-embedded tumour tissues were used for immunohistochemistry (IHC) (Hsu et al, 1981; Kayser and Gabius, 1997). Briefly, lung tumour tissue sections were deparaffinized by xylene (2 × 5 min) and rehydrated through alcohol 99% (2 × 5 min), 96% (1 × 2 min), 70% (1 × 2 min), 50% (1 × 2 min) to phosphate-buffered saline (PBS), pH 7.4 (3 × 3 min). After blocking: (i) endogenous peroxidase with 2% methanolic hydrogen peroxide for 15 min, (ii) non-specific binding sites with 5% normal goat serum in PBS for 30 min at room temperature, tissue sections were rinsed in PBS (3 × 3 min) and incubated with sheep IgG anti-cath B (KRKA) 1:200 diluted in PBS containing 5% normal goat serum in a humid chamber at room temperature overnight. After washing in PBS (3 × 3 min), tissue sections were incubated with biotinylated rabbit anti-sheep antibody (Vector Laboratories, Serva Heidelberg,

**Table 2** Immunohistochemical analysis of cathepsin B in lung tumour tissue

| | | Cath $B_{T-I}$[b] (no vs yes) Tumour | |
|---|---|---|---|
| | *n*[a] | No | Yes |
| Primary tumours (total) | 239 | 109 | 130 |
| SCC | 131 | 53 | 78 |
| AC | 108 | 56 | 52 |
| pT1 | 35 | 20 | 15 |
| pT2 | 144 | 67 | 77 |
| pT3 | 33 | 12 | 21 |
| pT4 | 23 | 7 | 16 |
| pN0 | 95 | 45 | 50 |
| pN1 | 71 | 33 | 38 |
| pN2 | 50 | 21 | 29 |
| pN3 | 19 | 7 | 12 |
| pTNM I | 80 | 40 | 40 |
| pTNM II | 41 | 16 | 25 |
| pTNM IIIa | 64 | 31 | 33 |
| pTNM IIIb | 32 | 9 | 23 |
| pTNM IV | 18 | 10 | 8 |

[a]Addition of subgroups is not equal to 239 and 147, because four TNMx categories could not be assessed. [b]92 lung tumour tissue sections consisting of 53 SCC and 39 AC out of 127 tissue samples were analysed in order to perform correlations with cath B assays.

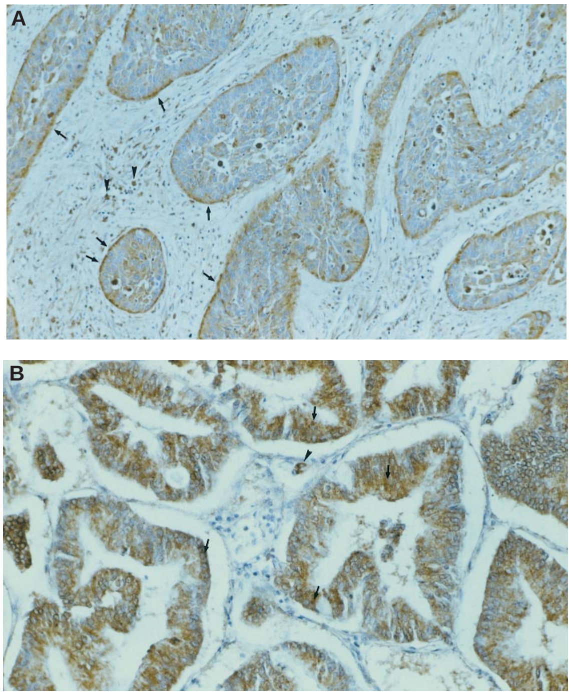

**Figure 1** Immunohistochemical staining of cath B in tissue sections derived from (**A**) a squamous cell carcinoma (SCC) and (**B**) an adenocarcinoma (AC). Magnification × 256. Immunostaining was performed by using a polyclonal antibody directed against cath B. The colour was developed with 3,3′-diaminobenzidine and hydrogen peroxide. This SCC sample represents the pattern observed in the majority of SCC sections with strongest immunoreactivity of cath B at the periphery of tumour cell foci embedded in the stroma (arrows). Cath B distribution in AC is a granular cytoplasmatic staining (arrows). Histiocytes were identified at higher magnification and marked by arrowheads

 *© 1999 Cancer Research Campaign*

**Table 3** Correlations

| | | Cath $B_{AT}$ Tumour/Lung | Cath $B_{A7.5}$ Tumour/Lung | Cath $B_C$ Tumour/Lung | Cath $B_{T-I}$ Tumour/Lung |
|---|---|---|---|---|---|
| SCC *n* = 65 | | | | | |
| Cath $B_{AT}$ | | – | – | – | – |
| Cath $B_{A7.5}$ | *r* | 0.64/0.75 | – | – | – |
| | *p* | < 0.001/< 0.001 | – | – | – |
| Cath $B_C$ | *r* | 0.25/0.61 | 0.24/0.61 | – | – |
| | *P* | < 0.05/< 0.001 | < 0.1/< 0.001 | – | – |
| Cath $B_{T-I}$[a] | *P* | 0.9/– | 0.9/– | 0.46/– | – |
| AC *n* = 59 | | | | | |
| Cath $B_{AT}$ | | – | – | – | – |
| Cath $B_{A7.5}$ | *r* | 0.39/0.78 | – | – | – |
| | *P* | < 0.01/< 0.001 | – | – | – |
| Cath $B_C$ | *r* | 0.55/0.78 | 0.67/0.79 | – | – |
| | *P* | < 0.001/< 0.001 | < 0.001/< 0.001 | – | – |
| Cath $B_{T-I}$[a] | *P* | < 0.05/– | < 0.01/– | < 0.1/– | – |

[a]Homogeneous variables, cath $B_{AT}$, cath $B_{A7.5}$, cath $B_C$ were recorded into two groups based on the immunohistochemical evaluation (cath $B_{T-I}$) negative and positive, respectively. To identify a relationship between the two new groups of cath $B_{AT}$, cath $B_{A7.5}$, cath $B_C$ we used Pearson's $\chi^2$ and Fisher's exact test.

Germany) in PBS with 5% normal goat serum for 30 min and with the Vectastain ABC Reagent (Vector) for 30 min. Peroxidase activity was developed with 3,3′-diaminobenzidine tetrahydrochloide (0.3 mg $ml^{-1}$ in 0.05 M Tris-buffer, pH 7.5), including 0.2% hydrogen peroxide for 10–15 min. Finally, tissue sections were light counterstained with 5% Harris's haematoxylin, dehydrated and mounted.

For IHC, the following control assays were performed: (i) incubation as in the assay, omitting the primary antibody; (ii) incubation omitting secondary antibody. Tissue sections were inspected by two independent pathologists (EK and KK). Both pathologists knew neither the diagnosis of the patients nor the result of the evaluation from each other. Both evaluations agree to 93%. Residual 7% were re-examined. The IHC scoring was performed separately for cath B in tumour cells (cath $B_{T-I}$) and cath B in histiocytes (cath $B_{H-I}$). Sections were classified into two groups according to the number of positively stained tumour cells or histiocytes and were considered positive for cath $B_{T-I}$ or cath $B_{H-I}$ when > 5% of stained cells were present. The intensity of staining was not considered as we observed that the intensity parallels the frequency (percentage) of immunopositive cells, similar as already reported (Castiglioni et al, 1994).

### Statistical analyses

To compare data of pairs of tumour and lung tissue, we used the two-tailed Wilcoxon's rank test. Differences in cath B levels in different groups of patients were tested by Mann–Whitney and Kruskal–Wallis test. The correlation between activities (cath $B_{AT}$, cath $B_{A7.5}$) and concentration (cath $B_C$) was calculated by non-parametric regression analysis and the significance of the Spearman rank correlation coefficient was evaluated by analysis of variance (ANOVA). Homogeneous variables, cath $B_{AT}$, cath $B_{A7.5}$, cath $B_C$, were recoded into two new groups based on the immunohistochemical evaluation cath $B_{T-I}$: negative and positive respectively. For correlation between these two new groups of cath $B_{AT}$, cath $B_{A7.5}$, cath $B_C$ and between the IHC scoring with different pTNM categories, Pearson's $\chi^2$ and Fisher's exact test were used. Univariate analysis of survival probability was performed by Kaplan and Meier analysis (1958), using the log-rank test for determination of statistical significance between the survival curves and multivariate analysis was performed using the Cox proportional hazard model (Cox, 1972). The discrimination levels of differentiate between subgroups of patients were calculated by the Critlevel programme (Abel et al, 1984). Various statistical packages (PC-Statistik, TOPSOFT, Hannover, Germany; Statistica, StatSoft, Hamburg, Germany) were applied.

**Table 4** Univariate survival analyses

| Variable | Histology | Unfavourable vs favourable characteristics | *P*-value |
|---|---|---|---|
| pTNM-staging | NSCLC | TNM III b, IV vs TNM I, II, III a | < 0.0001 |
| Cath $B_{T-I}$ | NSCLC | (+) vs (–) | < 0.0001 |
| Cath $B_{T-I}$ | SCC | (+) vs (–) | 0.00017 |
| Cath $B_{H-I}$ | SCC | (+) vs (–) | 0.028 |
| Cath $B_{T-I}$ | AC | (+) vs (–) | 0.039 |
| Cath $B_{H-I}$ | AC | (+) vs (–) | 0.05 |
| Cath $B_{A7.5}$ | SCC | > 292 vs < 292 (µEU $mg^{-1}$)[a] | 0.037 |
| Cath $B_{A7.5}$ | AC | > 292 vs < 292 (µEU $mg^{-1}$)[a] | 0.98 |
| Cath $B_{AT}$ | SCC | > 1674 vs < 1674 (µEU $mg^{-1}$)[a] | 0.19 |
| Cath $B_{AT}$ | AC | > 1674 vs < 1674 (µEU $mg^{-1}$)[a] | 0.91 |
| Cath $B_C$ | SCC | > 512 vs < 512 (ng $mg^{-1}$)[a] | 0.31 |
| Cath $B_C$ | AC | > 512 vs < 512 (ng $mg^{-1}$)[a] | 0.93 |
| Age | SCC | > 60 years vs < 60 years | 0.6 |
| Sex | SCC | Male vs Female | 0.23 |

[a]Cut-off values were calculated by a computer program developed by Abel et al (1984).

### RESULTS

### Distribution of cathepsin B in lung tumours

We measured enzymatic activities of total cath B at pH 6.0 (cath $B_{AT}$) and stable, active fraction at pH 7.5 (cath $B_{A7.5}$) as well as the concentration of the enzyme (cath $B_c$) in tissue extracts of 127 samples of NSCLC tumours and the corresponding non-tumourous lung parenchyma. A summary of the data is given in Table 1. The median levels of the enzymatic activities of cath $B_{AT}$,

*© 1999 Cancer Research Campaign* 

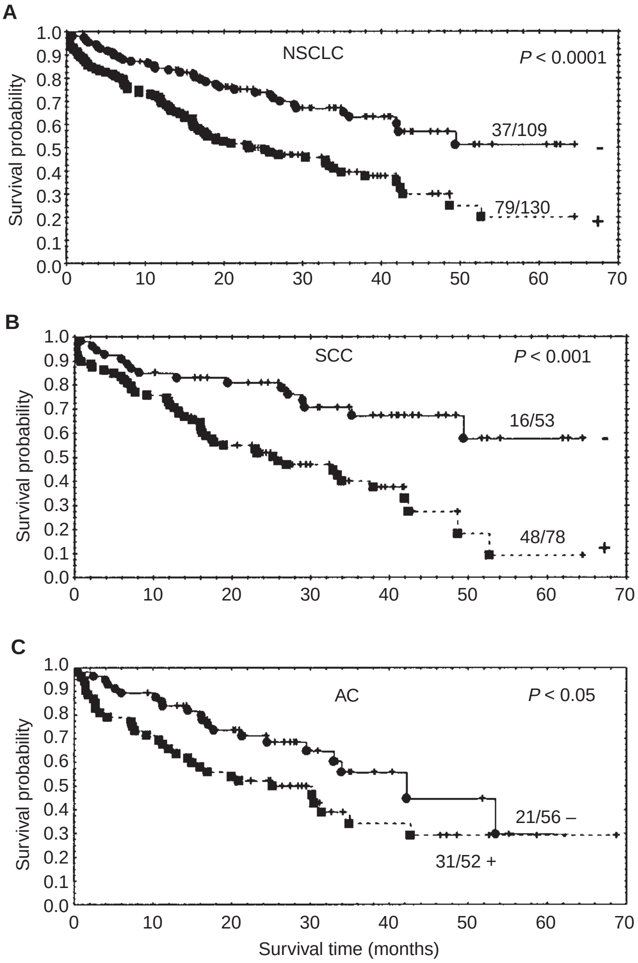

**Figure 2** Prognostic significance of tumour-associated cath B staining (cath $B_{T-I}$) for overall survival of patients. (**A**) Diagnosed for NSCLC, (**B**) diagnosed for SCC and (**C**) diagnosed for AC. Immunoreactivity of cath B was evaluated by two pathologists and scored as negative (< 5% positive tumour cells) and positive (> 5% positive tumour cells). In NSCLC 79 out of 130 (A+), in SCC 48 out of 78 (B+), and in AC 31 out of 52 (C+) patients with positive in cath $B_{T-I}$ had a significantly shorter overall survival (—■— deceased patients (uncensored data), —|— still living patients (censored data)) than 37 out of 109 NSCLC- (A–), 16 out of 53 SCC- (B–) and 21 out of 56 AC- (C–) patients with negative cath $B_{T-I}$ (–●– deceased patients, –|– still living patients)

cath $B_{A7.5}$ and the cath $B_c$ are 5.6-, 3.2- and 9.1-fold higher in tumour than in non-tumour lung parenchyma. The significance ($P < 0.001$) between tumour and normal tissue is high, although we observed certain overlapping in the activities and the concentration levels between tumour and non-tumour tissues. By taking the 99% percentile of the normal level of cath $B_{AT}$ (1278 [μEU $mg^{-1}$ $protein^{-1}$]), cath $B_{A7.5}$ (923 [μEU $mg^{-1}$ $protein^{-1}$]) and cath $B_C$ (512 [ng $mg^{-1}$ $protein^{-1}$]), considered as the upper limit of 'normal', we found an overlap with 52%, 80%, but only 7% of the respective tumour cases. Therefore, best discrimination between tumour and control tissue is indicated by increased cath B concentrations with a sensitivity of 93% and with a specificity of 99%.

For an IHC analysis (Table 2) we combined 92 out of the 127 samples where the cath B assays were performed with a second independent group of 147 samples, comprising the total of 239 NSCLC samples. According to histology, 131 of these patients suffered from squamous cell carcinoma (SCC) and 108 patients from adenocarcinoma (AC). Immunostaining for cath B was positive in 130 out of 239 of tissue sections (54.4%).

All these data were analysed with respect to tumour histology and pTNM-staging (as shown on Tables 1 and 2). There was no significant difference in the median levels of both types of cath B activities and cath $B_C$ between SCC and AC. We did not find a correlation with tumour size (pT) while cath $B_{AT}$, cath $B_{A7.5}$ and cath $B_C$ in primary tumours correlated very strongly with lymph node metastasis (pN status). These three parameters were significantly higher ($P < 0.01$, $P < 0.05$ and $P < 0.01$ respectively) in primary tumours of patients with lymph node metastases stage pN3 compared to the primary tumours without lymph node metastases (pN0). A steady increase of the median level of cath $B_{A7.5}$ activity was also observed from pN0 over pN1, pN2 to pN3 tumours, which was close to significance ($P = 0.073$). Significant association between cath $B_C$ and pTNM stages was observed, as the median value of cath $B_C$ was significantly ($P < 0.01$) higher in pTNM IIIb than pTNM I tumours, although not in pTNM IV tumours.

Table 2 shows that a clear positive immunoperoxidase reaction, associated with tumour cells, was observed among 131 SCC sections (59.5%) and 48.2% out of the 108 AC sections. However, there were significant differences in the staining patterns. In AC samples, the staining was distributed over the cytoplasm (Figure 1B) while in SCC samples, the intensity of immunoreactive cath B was strongest at the periphery of tumour cell foci embedded in non-tumour cell material (Figure 1A). Inflammatory cells such as histiocytes were always found associated with lung tumour cells and stained positively for cath B (cath $B_{H-I}$) in 102 out of 131 sections (77.9%) of SCC samples and in 75 out of 108 AC sections (69.5%). Cath $B_{T-I}$ was found to be more frequently positive in pT4- than in pT1-tumours and was more frequently positive in ΣpN1/pN2/pN3 than in pN0 tumours. Similar as for the total cath B concentration, positive labelling of tumour cell associated cath B (cath $B_{T-I}$) was observed in higher pTNM stages, as pTNM IIIb tumours were more frequently labelled than the pTNM I tumours ($P < 0.05$).

### Correlation

The correlation's between evaluated cath B parameters are listed in Table 3. In contrast to SCC we found in AC a positive correlation between all levels of cath B expression, cath B activities (cath $B_{AT}$, cath $B_{A7.5}$), cath B concentration and cath $B_{T-I}$ ($P < 0.05$, $P < 0.01$, and $P < 0.1$, respectively). In addition, the correlation in AC between cath $B_{AT}$, cath $B_{A7.5}$ and cath B concentration (both $P < 0.001$) was stronger compared to SCC ($P < 0.05$ and $P < 0.1$, respectively). In lung parenchyma of patients with SCC as well as with AC there was a significant correlation between cath B activities and cath B concentration (in all cases $P < 0.001$ and $P < 0.001$).

### Association of cath B activities, concentrations and immunostaining with survival probability

For prognosis of patients with NSCLC the histological cell type and the stage of the disease are the most important prognostic factors used in the clinical routine. In order to evaluate prognostic value of cath B alone and in comparison with the established clinical prognostic factors, we performed univariate and multivariate survival analyses, respectively.

© 1999 Cancer Research Campaign

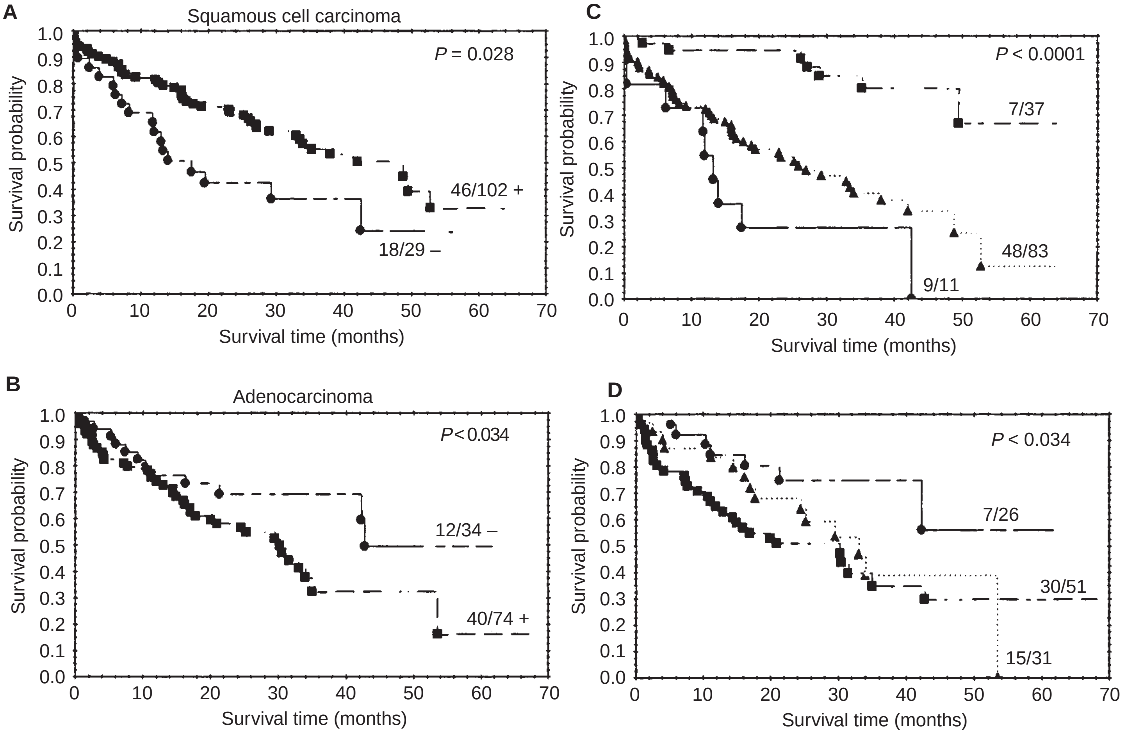

**Figure 3** Prognostic significance of immunohistochemical analysis of cath B in tumour cells (cath $B_{T-I}$) and in histiocytes (cath $B_{H-I}$) for overall survival. (**A**) SCC-patients with negative cath $B_{H-I}$ (–●–, –) have a significantly shorter overall survival than SCC patients with positive cath $B_{H-I}$ (—■—, +). (**B**) AC patients with positive cath $B_{H-I}$ had the opposite impact on survival. (**C**) Combination of cath $B_{T-I}$ and cath $B_{H-I}$ for SCC-patients. Nine out of 11 patients with positive cath $B_{T-I}$ but negative cath $B_{H-I}$ died in a 5-year observation period (–●–) but only seven out of 37 patients with negative cath $B_{T-I}$ and positive cath $B_{H-I}$ – expression died (—■—), the difference being highly significant ($P < 0.0001$). Patients with either positive/positive or negative/negative reaction in both cell types, were grouped together and show an intermediate survival rate (•••▲•••). (**D**) Combination of cath $B_{T-I}$ and cath $B_{H-I}$ for AC patients. Seven out of 26 patients with negative cath $B_{T-I}$ and negative cath $B_{H-I}$ died in a 5-year observation period (–●–) and 30 out of 51 patients with positive cath $B_{T-I}$ and positive or negative cath $B_{H-I}$ – expression died (—■—), the difference being highly significant ($P = 0.014$). The group with negative cath $B_{T-I}$ but positive cath $B_{H-I}$ (15/31) had an intermediate survival probability (•••▲•••). Comparison of all three groups by log-rank-test revealed a significance of $P = 0.034$

**Table 5** Cox regression analyses

| **Variable** | **Unfavourable vs favourable characteristics** | **Relative risk** | ***P*-value** |
|---|---|---|---|
| Multivariate I (NSCLC) | | | |
| pTNM-staging | TNM III b, IV vs TNM I, II, IIIa | 2.88 (2.4–3.5) | < 0.00001 |
| Cath $B_{T-I}$ | (+) vs (–) | 1.88 (1.5–2.3) | 0.0018 |
| Multivariate II (SCC) | | | |
| pTNM-staging | TNM III b, IV vs TNM I, II, IIIa | 3.33 (2.5–4.4) | < 0.000021 |
| Cath $B_{T-I}$ | (+) vs (–) | 2.43 (1.8–3.3) | 0.0028 |
| Multivariate III (SCC) | | | |
| pTNM-staging | TNM III b, IV vs TNM I, II, IIIa | 2.71 (1.8–4.1) | 0.012 |
| Cath $B_{A7.5}$ | > 292 vs < 292 (µEU $mg^{-1}$) | 2.31 (1.6–3.4) | 0.032 |
| Multivariate IV (AC) | | | |
| pTNM-staging | TNM III b, IV vs TNM I, II, IIIa | 2.54 (1.9–3.4) | 0.0014 |
| Cath $B_{T-I}$ | (+) vs (–) | 1.56 (1.2–2.1) | 0.12 |

### Univariate analyses

Table 4 presents the significance of prognosis for cath B variables in comparison to the age and sex of the patients and for two different histologies of lung tumours. In tumour homogenates cath $B_{AT}$ and cath $B_C$ showed no significant prognostic value for 5-year survival (Table 4). Noteworthy, for total population of NSCLC patients and for SCC patients cath $B_{AT}$ and cath $B_{A7.5}$ were of prognostic significance in short observation periods of 6 months (NSCLC: cath $B_{AT}$, $P < 0.05$) or 15 months (NSCLC and SCC: $P < 0.05$ and $P < 0.05$ respectively, data not shown). However, IHC analysis revealed that tumour cell associated cath B (cath $B_{T-I}$) was of prognostic significance for 5-year survival in the total population of NSCLC cancer patients ($n = 239$, $P < 0.0001$). Comparing the two different histologies cath $B_{A7.5}$ and cath $B_{T-I}$ were also of prognostic significance for SCC patients ($n = 65$, $P < 0.05$; $n = 131$, $P < 0.001$, respectively), as illustrated in Figure 2. In AC

*© 1999 Cancer Research Campaign*

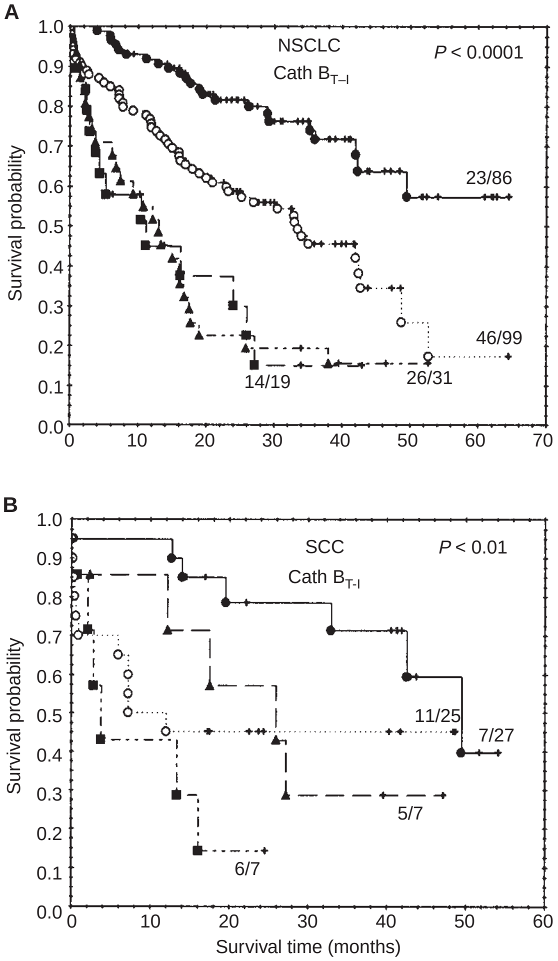

**Figure 4** Survival analyses of patients stratified by pTNM stage and by cath $B_{T-I}$ and cath $B_{A7.5}$: (**A**) pTNM-stage and cath $B_{T-I}$ for the total of 235 NSCLC patients. (**B**) pTNM stage and cath $B_{A7.5}$ for 131 SCC patients. In NSCLC- and SCC patients the combination of pTNMI–IIIa and negative cath $B_{T-I}$ (23/86) or low (< 292 µEU $mg^{-1}$) cath $B_{A7.5}$ (7/27)(–●–) is significantly different from pTNM I–IIIa and positive cath $B_{T-I}$ (46/99) or high (> 292 µEU $mg^{-1}$) cath $B_{A7.5}$ (11/25) (•••○•••). No significant difference was observed between NSCLC-patients with pTNM stage IIIb and IV and negative cath $B_{T-I}$ (—▲—), 26/31) compared with positive cath $B_{T-I}$ (–■–, 14/19). A significant difference could be observed between SCC patients with high pTNM stage IIIb, IV and low cath $B_{A7.5}$ (–▲–, 5/7) compared with high cath $B_{A7.5}$ (—■—, 6/7)

patients ($n$ = 108, $P$ < 0.05), only cath $B_{T-I}$ was significant. The IHC analysis also revealed that cath B in tumour associated histiocytes (cath $B_{H-I}$) in SCC and AC had different prognostic impact for survival (Figure 3 A,B). SCC patients with negative cath $B_{H-I}$ had significantly shorter overall survival than SCC patients with positive cath $B_{H-I}$ ($P$ = 0.028), while AC patients with positive cath $B_{H-I}$ had shorter survival rate ($P$ < 0.05).

Furthermore, the combination of positive cath B in tumours and cath B in tumour associated histiocytes was also of prognostic relevance discriminating between additional subgroups of patients (Figure 3 C,D). Nine out of 11 SCC patients with positive cath $B_{T-I}$ but negative cath $B_{H-I}$ died within 5 years (median survival time 13.3 months). In contrast, in the group of patients with negative cath $B_{T-I}$ and positive cath $B_{H-I}$ only 7 out of 37 patients died in this period. The median survival time in this group was 35 months ($P$ < 0.0001). Patients with either positive cath $B_{T-I}$ and positive cath $B_{H-I}$ or negative cath $B_{T-I}$ and negative cath $B_{H-I}$ showed an intermediate value and were therefore grouped together (the median survival time was 20.7 months). In contrast to this, in AC patients, the best survival was observed in the group with negative cath $B_{T-I}$ and negative cath $B_{H-I}$: 7 out of 26 patients died in 5 years; the median/mean survival time was 21.5/26.2 months respectively. The patients with positive cath $B_{T-I}$ and positive or negative cath $B_{H-I}$ were grouped together: 30 out of 51 patients died in 5 years, the median/mean survival time was 22.5/21.9 months respectively. The group with negative cath $B_{T-I}$ and positive cath $B_{H-I}$ showed a median/mean survival time of 24.5/21.9 months respectively. The probability of survival between these groups differed significantly ($P$ = 0.034).

### Multivariate analyses

In the multivariate analysis, only factors with a significant relationship to the survival probability found by the univariate analysis and independent from other factors were included. Thus, only two factors were considered: pTNM-staging and either cath $B_{T-I}$ as shown on Table 5 (Multivariate I, II, IV) or cath $B_{A7.5}$ (Table 5, Multivariate III). In the total population of NSCLC patients and in patients with SCC, only pTNM staging and cath $B_{T-I}$ or cath $B_{A7.5}$ were significant for prognosis. Cath B had no impact on prognosis in patients with AC.

As demonstrated in Table 5, pTNM-staging remained the best independent prognostic factor for patients with SCC and with AC. In addition, in SCC patients only, cath $B_{T-I}$ and cath $B_{A7.5}$ were powerful independent prognostic factors.

To answer the question whether a combination of independent prognostic factors cath $B_{T-I}$, cath $B_{A7.5}$ and the pTNM-staging provide new information, patients were subdivided into two groups consisting of patients in a low pTNM-stage (pTNM I–IIIa) and a high pTNM-stage (pTNM IIIb and IV). Kaplan–Meier analyses showed that only 23 out of 86 patients (Figure 4A) in pTNM I–IIIa and negative cath $B_{T-I}$ compared to 46 out of 99 patients with pTNM I–IIIa and positive cath $B_{T-I}$ died within 5 years. The median survival time were 29.1 and 24.0 months. Comparable results were obtained in patients with SCC, since only seven out of 27 patients with low cath $B_{A7.5}$ activities (values < 292 [µEU $mg^{-1}$]) in pTNM I–IIIa compared to 11 out of 25 patients with cath $B_{A7.5}$ activity values above this cut-off point in pTNM I–IIIa died within 5 years. The median survival times were 36.8 and 12.0 months. For NSCLC-patients in high stage pTNM IIIb and IV, cath $B_{T-I}$ did not further discriminate between good and poor survival and both groups have the worst survival probability. This is in contrast to patients with SCC, where patients in pTNM-stage IIIb and IV and cath $B_{A7.5}$ values below 292 [µEU $mg^{-1}$] have a better overall survival probability compared to those patients above this cut-off point.

### DISCUSSION

Previous studies on cath B in lung tumours, using either homogenates (Krepela et al, 1990; Sedo et al, 1991; Ebert et al, 1994; Ledakis et al, 1996; Werle et al, 1997*a*, 1997*b*) or IHC analysis of cath B (Higashiyama et al, 1991, 1993; Ozeki et al, 1993; Inoue et al, 1994; Sukoh et al, 1994*a*, 1994*b*), showed significantly enhanced expression of the enzyme at activity and protein levels in patients suffering from NSCLC. In this study, we clearly confirmed our earlier results as we found 9.1-fold increased cath B protein concentration as well as 5.6-fold increased total cath B activity and 3.2-fold increase in the fraction

 *© 1999 Cancer Research Campaign*

of cath B activity, stable at pH 7.5 (Werle et al, 1997*b*) in tumour compared with normal lung tissue homogenates. Our data concerning enzyme expression revealed by IHC agree with previous investigations (Higashiyama et al, 1991, 1993; Ozeki et al, 1993; Inoue et al, 1994; Sukoh et al, 1994*a*, 1994*b*) demonstrating that more elevated levels of cath B were found in primary tumours, which invaded into lymph nodes. Noteworthy, fraction of cath B activity stable at pH 7.5 (cath $B_{A7.5}$) steadily increased in more malignant lung tumours (from pN1 to pN3). These data strongly support the association of cath B with the pathobiochemical mechanism of tumour progression.

In relation to prognosis, the histological type and the tumour stage (TNM) of the lung tumours are considered as key factors relevant to prognosis for patients with NSCLC. However, new prognostic factors may still be needed to discriminate the patients with worse prognosis in low risk, e.g. low TNM-stage tumours. In this study, we found that tumour stage is still the best prognostic factor, but in addition, we demonstrated that immunohistochemical analysis of cath B, in particular tumour cell-associated cath B (cath $B_{T-I}$) is also a powerful independent prognostic factor in NSCLC patients. In contrast, cath B activities (cath $B_{AT}$, cath $B_{A7.5}$) and concentration (cath $B_C$), measured in tumour homogenates, had no prognostic relevance in the total population of NSCLC patients in a 5-year observation period. This is in contrast to the study by Ebert et al (1994) and Knoch et al (1994), but similar to the recent study (Werle et al, 1997*a*). The discrepancy with earlier studies is most likely due to longer observation period of 5 years in the present study. It is interesting to note that cath $B_{AT}$ and cath $B_{A7.5}$ were of prognostic significance in shorter observation period of 6 months (cath $B_{AT}$, $P < 0.05$; Ebert et al, 1994) and 15 months (both $P < 0.05$; unpublished data). When different histologies were considered separately, prognostic relevance of cath $B_{AT}$ and cath $B_{A7.5}$ were found for patients suffering from SCC, already in shorter observation period (Werle et al, 1997*a*). In contrast, in AC no prognostic impact of cath $B_{AT}$, cath $B_{A7.5}$ and cath $B_C$ was found in short (6, 15 and 24 months) and long (5 years) observation period.

Our findings agree with other univariate- and the multivariate survival analyses of cath B IHC analysis in tumour cells of adenocarcinoma (Sukoh et al, 1994*a*, 1994*b*). Moreover, Ozeki et al (1993) further differentiated AC patients and demonstrated prognostic significance of cath B for lung AC of the Clara cell type only, but not for other subtypes of AC patients. The differences in these results may be due to different reagents and protocols used for IHC analysis and non-standardized scope of histological classifications of AC subtypes. It is known from large scale studies, that after surgery, the patients with SCC have a better overall survival probability than those with AC (Mountain, 1991). As indicated above, lung tumours of different histologies also differ with respect to cath B expression, distribution and relevance to prognosis. For example, in tumour homogenates, a fraction of cath B, stable at pH 7.5, cath $B_{A7.5}$ was a prognostic factor for SCC patients, but not for AC patients (Werle et al, 1997*a*). There was a significant correlation between cath $B_{AT}$, cath $B_C$ and cath $B_{T-I}$ in AC, which was not found in SCC tumours. Immunohistochemical analysis of tumour cell associated cath B also revealed that the distribution of cath B over the cytoplasm of the tumour cell in AC was homogenous, while in SCC cath B was highly concentrated at the periphery of tumour cell foci, indicating that this fraction of cath B was possibly involved in tumour cell invasion. In these tumours (AC), a significant difference for patients with positive and negative expression of cath B in tumour cells (cath $B_{T-I}$) was found by the univariate analysis, but not in multivariate survival analyses using Cox regression model. Although our results are in close agreement to large-scale studies of Sukoh et al (1994*a*, 1994*b*) and Ozeki et al (1993), there still exists a recent report from Mori et al (1997), who could not demonstrate a significant correlation between cath B overexpression and overall survival probability of 31 lung cancer patients in TNM stage I. The discrepancy between these studies might be due to an insufficient number of patients in the study of Mori et al (1997), distinct staining procedures, which included the use of divergent cath B antisera and different cut-off values for gradation of cath B stained tissue sections. However, cath B was also positive in 11 out of 12 SCC and 11 out of 15 AC tumours demonstrating an overexpression of cath B in lung tumours.

Immunohistochemistry also allows for a distinction between different cell types in tumour sections and we demonstrated a strong positive staining also in tumour-associated histiocytes, cath $B_{H-I}$. In SCC patients, the presence of cath B in histiocytes had an impact on longer survival and this effect was stronger in cath $B_{T-I}$-negative tumours. AC positive cath $B_{H-I}$ indicated worse prognosis, particularly in tumours with positive cath $B_{T-I}$ in tumour cells. These results indicate a possible role of cath B in tumour associated histiocytes. In general, the role of macrophages is still controversial. On one hand, activated macrophages are cytotoxic to tumour cells, as for example, interferon-gamma cured murine tumours in metastasis (Fidler, 1974), although the regression of primary tumours was not observed. Similarly, in-situ activated macrophages are involved in host resistance to lymphoma metastasis probably by a production of nitric oxide (Umansky et al, 1995). On the other hand, macrophages were found to directly stimulate tumour cell growth, inducing neovascularization and tissue dissolution. Furthermore somatic hybridization of tumour cells with the inflammatory macrophages was observed, resulting in highly metastatic variants of the original clones (Mantovani, 1990). We therefore suggest that different pathobiochemical mechanisms, involving cath B of macrophages, might be responsible for malignant progression of lung tumours of different histologies.

In conclusion, out of all cath B variables, only positive cath $B_{T-I}$ in NSCLC/SCC patients and high cath $B_{A7.5}$ in SCC patients had high impact on prognosis in a 5-year observation period. Furthermore, in low pTNM stages (I, II and IIIa) overexpression of cath $B_{T-I}$ in NSCLC patients and high cath $B_{A7.5}$ in SCC patients, indicated worse prognosis. Overexpression of cath $B_{H-I}$ in histiocytes might be useful in discriminating patients with higher risk for survival, depending on tumour histology.

### ACKNOWLEDGEMENTS

We thank Prof. Werner Ebert for many helpful discussions and support of this work, which was in most of its parts performed in his laboratory and Prof. Vito Turk (Jozef-Stefan Institute, Slovenia) for his continuous support and encouragement. We thank Wolfgang Klein for excellent technical assistance and Dr Hans Knoch, Britta Jülke, Clemens Kraft and Alexander Staib for providing tissue homogenates and for evaluation of clinical data of lung cancer patients, Dr Thomas Muley for purified alveolar macrophages (all from the Thoraxhospital Heidelberg) and Dr Werner Rittgen (Deutsches Krebsforschungszentrum) for his help in statistical evaluation. TL acknowledges a support from German

© 1999 Cancer Research Campaign

Cancer Center for the grant for short-term visits. This work was supported by grants of the Tumorzentrum Heidelberg-Mannheim, the European Community Concerted Action BIOMED-1 (BMH1-CT93-1346), the Bilateral German–Slovenian Research Project (SLO-027-95) and the Deutsche Krebshilfe – Dr Mildred-Scheel-Stiftung (10-1985-Eb 1) and by the Grants J3-6208, J1-0640 (to JK) and L3-7981 and L3-8919 (to TL) from the Ministry of Science and Technology of Republic of Slovenia.

### REFERENCES

Abel U, Berger J and Wiebelt H (1984) Critlevel. An exploratory procedure for the evaluation of quantitative prognostic factors. *Methods Inf Med* **23**: 154–156

Berquin IM and Sloane BF (1995) Cysteine proteases and tumor progression. *Perspect Drug Discovery Design* **2**: 371–388

Bradford MM (1976) A rapid and sensitive method for the quantitation of microgram quantities of protein utilizing the principle of protein-dye binding. *Anal Biochem* **72**: 248–254

Budihna M, Strojan P, Šmid L, Škrk J, Vrhovec I, Zupevc A, Rudolf Z, Zargi M, Krasovec M, Svetic B, Kopitar-Jerala N and Kos J (1996) Prognostic value of cathepsins B, H, L, D and their endogenous inhibitors stefins A and B in head and neck carcinoma. *Biol Chem Hoppe-Seyler* **377**: 385–390

Calkins CC and Sloane BF (1995) Mammalian cysteine protease inhibitors: biochemical properties and possible roles in tumor progression. *Biol Chem Hoppe-Seyler* **376**: 71–80

Castiglioni T, Merino MJ, Elsner B, Lah TT, Sloane BF and Emmert-Buck MR (1994) Immunohistochemical analysis of cathepsins D, B, and L in human breast cancer. *Hum Pathol* **25**: 857–862

Cox DR (1972) Regression models and life tables (with discussion). *J R Stat Soc* **187**: 187–220

Ebert W, Knoch H, Werle B, Trefz G, Muley Th and Spiess E (1994) Prognostic value of increased lung tumor tissue cathepsin B. *Anticancer Res* **14**: 895–900

Elliot E and Sloane BF (1996) The cysteine protease cathepsin B in cancer. *Perspect Drug Discovery Design* **6**: 12–32

Emmert-Buck MR, Roth MJ, Zhuang Z, Campo E, Rozhin J, Sloane BF, Liotta LA and Stetler-Stevenson WG (1994) Increased gelatinase A (MMP-2) and cathepsin B activity in invasive tumor regions of human colon cancer samples. *Am J Pathol* **145**: 1285–1290

Erdel M, Trefz G, Spiess E, Habermaas S, Spring H, Lah T and Ebert W (1990) Localization of cathepsin B in two human lung cancer cell lines. *J Histochem Cytochem* **38**: 1313–1321

Fidler IJ (1974) Inhibition of pulmonary metastasis by intravenous injection of specifically activated macrophages. *Cancer Res* **34**: 1074–1078

Hermanek P and Sobin L (1987) *TNM Classification of Malignant Tumors*, 4th edn. International Union against Cancer (UICC). Springer: Berlin

Higashiyama M, Doi O, Kodama K, Yokouchi H and Tateishi R (1991) The prognostic implications of the cathepsin B expression in pulmonary adenocarcinomas. An immunohistochemical study. (In Japanese) *Jpn J Cancer Clin* **37**: 1151–1157

Higashiyama M, Doi O, Kodama K, Yokouchi H and Tateishi R (1993) Cathepsin B expression in tumour cells and laminin distribution in pulmonary adenocarcinoma. *J Clin Pathol* **46**: 18–22

Honn KV, Timar J, Rozhin J, Bazaz R, Sameni M, Ziegler G and Sloane BF (1994) A lipoxygenase metabolite, 12-(S)-HETE, stimulates protein kinase C-mediated release of cathepsin B from malignant cells. *Exp Cell Res* **214**: 120–130

Hsu SM, Raine L, and Fanger H (1981) Use of avidin-biotin peroxidase complex (ABC) in immunoperoxidase techniques: a comparison between ABC and unlabeled antibody (PAP) procedures. *J Histochem Cytochem* **29**: 577–580

Hughes SJ, Glover TW, Zhu XX, Kuick R, Thoraval D, Orringer MB, Beer DG, Hanash S (1998) A novel amplicon at 8p22–23 results in overexpression of cathepsin B in esophageal adenocarcinoma. Proc Natl Acad Sci USA **95**: 12410–12415

Inoue T, Ishida T, Sugio K and Sugimachi K (1994) Cathepsin B expression and laminin degradation as factors influencing prognosis of surgically treated patients with lung adenocarcinoma. *Cancer Res* **54**: 6133–6136

Kaplan EL and Meier P (1958) Nonparametric estimation from incomplete observations. *J Am Stat Assoc* **53**: 457–481

Kayser K and Gabius H-J (1997) Graph theory and the entropy concept in histochemistry. Theoretical considerations, application in histopathology and the combination with receptor-specific approaches. In: *Progress in Histochemistry and Cytochemistry*, Graumann W, Bendayan M, Bosman FT, Heitz PhU, Larsson L-I, Wolfe HJ (eds), Vol. 32, Gustav Fischer: Stuttgart, Jena, Lübeck, Ulm

Knoch H, Werle B, Ebert W and Spiess E (1994) Imbalance between cathepsin B and cysteine proteinase inhibitors is of prognostic significance in human lung cancer. *Int J Oncol* **5**: 77–85

Kos J, ŠakSmid L, Krasovec M, Svetic B, Lenarcic B, Vrhovec I,Škrk J and Turk V (1995) Lysosomal proteases cathepsins D, B, H, L and their inhibitors stefins A and B in head and neck cancer. *Biol Chem Hoppe-Seyler* **376**: 401–405

Kos J, Štabuc B, Schweiger A, Krasovec M, Cimerman N, Kopitar-Jerala N and Vrhovec I (1997) Cathepsins B, H, L and their inhibitors stefin A and cystatin C in sera of melanoma patients. *Clin Cancer Res* **3**: 1815–1822

Kos J, Nielsen H-J, Krasovec M, Christensen IJ, Cimerman N, Stephens RW and Brünner N (1998) Prognostic values of cathepsin B and carcinoembryonic antigen in sera of patients with colorectal cancer. *Clin Cancer Res* **4**: 1511–1516

Krepela E, Kasafirek E, Novak K and Viklicky J (1990) Increased cathepsin B activity in human lung tumors. *Neoplasma* **37**: 61–70

Lah T and Kos J (1998) Cysteine proteinases in cancer progression and their clinical relevance for prognosis. *Biol Chem* **379**: 125–130

Lah TT, Kokalj-Kunovar M, Štrukelj B, Pungerèar J, Barliè-Maganja D, Drobniè-Košorok M, Kastelic L, Babnik J and Turk V (1992) Stefins and lysosomal cathepsins B, L and D in human breast carcinoma. *Int J Cancer* **50**: 36–44

Lah TT, Calaf G, Kalman E, Shinde BG, Somers R, Russo J, Jarocz D, Zabreckzy J and Daskal I (1995*a*) Cathepsins D, B and L in human breast carcinoma and in transformed cells. *J Biol Chem Hoppe-Seyler* **376**: 357–363

Lah TT, Hawlley M, Rock KL and Goldberg AL (1995*b*) Gamma-interferon causes selective induction of lysosomal proteases cathepsins B and L, in macrophages. *FEBS Letters* **262**: 85–89

Lah TT, Kos J, Blejec A, Frkoviè-Georgio S, Golouh R, Vrhovec I and Turk V (1997) The expression of lysosomal proteinases and their inhibitors in breast cancer: possible relationship to prognosis of the disease. *Pathology Oncology Research* **3**:(2), 89–98

Ledakis P, Tester WT, Rosenberg N, Romero-Fischmann D, Daskal I and Lah TT (1996) Cathepsins D, B, and L in malignant human lung tissue. *Clin Cancer Res* **2**: 561–568

Leto G, Tumminello FM, Pizzolanti G, Montalto G, Soresi M, Carroccio A, Ippolito S and Gebbia N (1997) Lysosomal aspartic and cysteine proteinases serum levels in patients with pancreatic cancer or pancreatitis. *Pancreas* **14**: 22–27

Manegold C and Drings P (1998) Chemotherapie des nichtkleinzelligen Lungenkarzinoms. In: *Thoraxtumoren: Diagnostik – Staging – gegenwärtiges Therapiekonzept*, Drings P, Vogt-Moykpf I (eds), pp. 310–327. Springer Verlag: Heidelberg, Germany

Mori M, Kohli A, Baker SP, Savas L, Fraire AE (1997) Laminin and cathepsin B as prognostic factors in stage I non-small cell lung cancer: are they useful? *Mod Pathol* **10**: 572–577

Mantovani A (1990) Tumour-associated macrophages. *Curr Opin Immunol* **2**: 689–692

Mikkelsen T, Yan PS, Ho KL, Sameni M, Sloane BF and Rosenblum ML (1995) Immunolocalization of cathepsin B in human glioma: implications for tumor invasion and angiogenesis. *Neurosurg* **83**: 285–290

Mountain CF (1991) Surgical treatment of lung cancer. *Critic Rev Oncol/Hematol* **11**: 179–207

Opdenakker G and Van Damme J (1992) Chemotactic factors, passive invasion and metastasis of cancer cells. *Immunol Today* **13**: 464–465

Ozeki Y, Takishima K, Takagi K, Aida S, Tamai S, Mamiya G and Oagata T (1993) Immunohistochemical analysis of cathepsin B expression in human lung adenocarcinoma: the role in cancer progression. *Jpn J Cancer Res* **84**: 972–975

Rempel SA, Rosenblum ML, Mikkelsen T, Yan PS, Ellis KD, Golembieski WA, Sameni M, Rozhin J, Ziegler G and Sloane BF (1994) Cathepsin B expression and localization in glioma progression and invasion. *Cancer Res* **54**: 6027–6031

Rozhin J, Robinson D, Stevens MA, Lah TT, Honn KV, Ryan RE and Sloane BF (1987) Properties of a plasma membrane-associated cathepsin B-like cysteine proteinase in metastatic B16 melanoma variants. *Cancer Res* **47**: 6620–6628

Rozhin J, Sameni M, Ziegler G and Sloane BF (1994) Pericellular pH affects distribution and secretion of cathepsin B in malignant cells. *Cancer Res* **54**: 6517–6525

Schraube P, Kimming B, Latz D, Flentje M and Wannenmacher M (1998) Radiotherapie des Bronchialkarzinoms. In: *Thoraxtumoren: Diagnostik – Staging – gegenwärtiges Therapiekonzept*, Drings P, Vogt-Moykopf I (eds), pp. 277–295. Springer Verlag: Heidelberg, Germany

© 1999 Cancer Research Campaign

Sedo A, Krepela E and Kasafirek E (1991) Dipeptidylpeptidase IV, prolylendopeptidase and cathepsin B activities in primary human lung tumors and lung parenchyma. *J Cancer Res Clin Oncol* **117**: 249–253

Shuja S, Sheahan K and Murnane MJ (1991) Cysteine endopeptidase activity levels in normal human tissues, colorectal adenomas and carcinomas. *Int J Cancer* **49**: 341–346

Sloane BF, Rozhin J, Hatfield JS, Crissman JD and Honn KV (1987) Plasma-membrane associated cysteine proteinases in human and animal tumors. *Exp Cell Biol* **55**: 209–224

Sloane BF, Moin K, Sameni M, Tait LR, Rozhin J and Ziegler G (1994) Membrane association of cathepsin B can be induced by transfection of human breast epithelial cells with c-Ha-ras oncogene. *J Cell Sci* **107**: 373–384

Stonelake PS, Jones CE, Neoptolemos JP and Baker PR (1997) Proteinase inhibitors reduce basement membrane degradation by human breast cancer cell lines. *Br J Cancer* **75**: 951–959

Strohmaier A-R, Porwol T, Acker H and Spiess E (1997) Tomography of cells by confocal laser scanning microscopy and computer-assisted three-dimensional image reconstruction: Localization of cathepsin B in tumor cells penetrating collagen gels in vitro. *J Histochem Cytochem* **45**: 975–983

Sukoh N, Abe S, Ogura S, Isobe H, Takekawa H, Inoue K and Kawakami Y (1994*a*) Immunohistochemical study of cathepsin B. Prognostic significance in human lung cancer. *Cancer* **74**: 46–51

Sukoh N, Abe S, Nakajima I, Ogura S, Isobe H, Inoue K and Kawakami Y (1994*b*) Immunohistochemical distributions of cathepsin B and basement membrane antigens in human lung adenocarcinoma: association with invasion and metastasis. *Virchows Arch* **424**: 33–38

Trefz G, Lüthgens K, Erdel M, Spiess E and Ebert W (1989) Plasminogen activator and cathepsin B in normal and malignant human lung tissue. *J Cancer Res Clin Oncol* **115**: S50

Ulbricht B, Hagmann W, Ebert W and Spiess E (1996) Differential secretion of cathepsins B and L from normal and tumor human lung cells stimulated by 12(S)-hydroxy-eicosatetraenoic acid (12(S)-HETE). *Exp Cell Res* **226**: 255–263

Umansky V, Rocha M, Kruger A, van Hoegen P and Schirrmacher V (1995) In situ activated macrophages are involved in host resistance to lymphoma metastasis by production of nitric oxide. *Int J Cancer* **7**: 33–40

Werle B, Ebert W, Klein W and Spiess E (1994) Cathepsin B in tumors, normal tissue and isolated cells from the human lung. *Anticancer Res* **14**: 1169–1176

Werle B, Ebert W, Klein W and Spiess E (1996) Charge polymorphism in human lung cell procathepsin B. *Anticancer Res* **16**: 49–54

Werle B, Jülke B, Lah T, Kos J, Spiess E and Ebert W (1997*a*) Cathepsin B and cathepsin L: prognostic factors in human lung cancer? In *Proteolysis in Cell Function*, Hopsu-Havu M, Jarvinen M, Kirschke H (eds), pp. 472–478. IOS Press: Amsterdam, The Netherlands

Werle B, Jülke B, Lah T, Spiess E and Ebert W (1997*b*) Cathepsin B fraction active at physiological pH of 7.5 is of prognostic significance in squamous cell carcinoma of human lung. *Br J Cancer* **75**: 1137–1143

World Health Organization (1981) *Histological Classification of Lung Tumors*. WHO: Geneva

Yan SQ, Sameni M and Sloane BF (1998) Cathepsin B and human tumor progression. *Biol Chem* **379**: 113–123

*© 1999 Cancer Research Campaign*
